# Insights into morbidity and mortality in Gaucher disease: a cohort study

**DOI:** 10.1016/j.ymgmr.2026.101369

**Published:** 2026-10-17

**Authors:** Lucas Ferreira Teixeira, Fabiano Poswar, Dévora Natalia Randon, Amanda Pasqualotto, Ida Vanessa Doederlein Schwartz

**Affiliations:** a School of Medicine, Universidade Federal do Rio Grande do Sul (UFRGS), Porto Alegre, Brazil; b National Institute for Rare Diseases (InRaras), Porto Alegre, Brazil; c Medical Genetics Service, Hospital de Clínicas de Porto Alegre (HCPA), Porto Alegre, Brazil; d Postgraduate Program in Genetics and Molecular Biology, UFRGS, Porto Alegre, Brazil; e Basic Research and Advanced Investigations in Neurosciences (BRAIN) Laboratory, Experimental Research Center, HCPA, Porto Alegre, Brazil; f Universidade do Vale do Rio dos Sinos (UNISINOS), São Leopoldo, RS, Brazil; g Department of Genetics, UFRGS, Porto Alegre, Brazil

**Keywords:** Gaucher disease, Lysosomal storage disorder, Enzyme replacement therapy, Malignancy, Comorbidity, Mortality

## Abstract

**Background:**

Gaucher disease (GD) is a rare lysosomal disorder classified, on the basis of neurological involvement, into type 1 (GD1, non-neuronopathic) and types 2 (GD2) and 3 (GD3) (neuronopathic). Data on morbidity and mortality in Latin American cohorts remain scarce.

**Aims:**

To characterise morbidity and mortality patterns in a Brazilian GD cohort.

**Methods:**

Retrospective cohort study at a single GD referral centre in Porto Alegre, Brazil (2003–2023; *n* = 53; GD1 = 45, GD2 = 4, GD3 = 4). Diagnosis was molecularly confirmed by Sanger and massive parallel sequencing of *GBA1*. Comorbidities and causes of death were retrieved by chart review; survival was estimated by Kaplan–Meier analysis. Results: Median follow-up was 15 years. In GD1, comorbidities were predominantly psychiatric (71%), metabolic/endocrinological (69%) and cardiovascular (60%). Nine patients (17%) died: four GD1 patients (median age 65 years; three from malignancy, one from sepsis), all four GD2 patients (median age 5 months; respiratory or hepatic failure, sepsis) and one GD3 patient (Sudden Unexpected Death in Epilepsy at 27 years).

**Conclusions:**

The phenotype-specific mortality pattern — malignancy as the leading cause of death in GD1, uniform early death in GD2 reflecting the disease's neurological trajectory rather than a failure of enzyme replacement therapy, and neurological death in GD3 — underscores the substantial burden of oncological, cardiovascular, metabolic and neurological comorbidities in this population and supports systematic multidisciplinary surveillance as a routine component of GD care in Brazil, even in resource-limited settings.

**Take-home message:**

Adult patients with Gaucher disease type 1 require close clinical monitoring for oncological, cardiovascular, metabolic and psychiatric comorbidities. Gaucher disease type 2 remains a devastating disease with a poor prognosis under current therapies.

## Highlights

• Retrospective single-centre Brazilian Gaucher disease cohort of 53 patients.
• GD1 mortality was driven by malignancy despite enzyme replacement therapy.
• All GD2 patients died before 15 months of age despite ERT in most cases.
• GD3 death occurred as Sudden Unexpected Death in Epilepsy at 27 years.

## 1 Introduction

Gaucher disease (GD) is a rare inborn error of metabolism, inherited in an autosomal recessive manner. Pathogenic variants in the *GBA1* gene (OMIM #606463) impair the activity of the lysosomal enzyme acid β-glucocerebrosidase (GCase; EC 3.2.1.45), leading to accumulation of glucosylceramide and glucosylsphingosine within the lysosomes of macrophages in the spleen, bone marrow, liver and central nervous system. The clinical phenotype and disease severity are highly variable, reflecting the combined influence of genetic variants, residual enzyme activity, age at onset and time on treatment [1], [2].

GD has historically been classified into three subtypes on the basis of neurological involvement. Type 1 (GD1) is non-neuronopathic, characterised by visceral glucocerebroside storage in the spleen, liver and bone marrow. Type 2 (GD2) presents with acute-onset neurological symptoms alongside visceral disease and is usually fatal before 2 years of age. Type 3 (GD3) combines visceral accumulation of glucocerebroside — with splenic, hepatic, bone-marrow and pulmonary involvement — with chronic, progressive neurological impairment that compromises functional status; survival is longer than in GD2 but remains reduced. Current evidence supports a view of GD as a clinical and phenotypic continuum shaped by specific genetic variants and individual biological susceptibility [3].

Disease-specific therapies for GD — enzyme replacement therapy (ERT) and substrate reduction therapy (SRT) — have well-documented efficacy in improving clinical outcomes and reducing mortality [4]. Emerging therapeutic approaches include ambroxol for the neurological manifestations of GD3 [5] and gene-therapy strategies: several adeno-associated virus (AAV)–based programmes targeting *GBA1* are in preclinical or early-phase development, and GALILEO-3 (FLT201) is currently the only phase-3 trial [6]. Beyond these advances, a broad clinical evaluation of comorbidities remains essential: characterising morbidity and mortality patterns in patients treated with ERT or SRT is critical for understanding disease progression and optimising therapeutic strategies. The social and environmental context in which patients live — including access to specialised care, socioeconomic conditions and geographic location — is a further determinant of long-term outcomes, an issue particularly relevant for patients with rare diseases in low- and middle-income countries [7]. Within this context, we conducted an evaluation of comorbidities and mortality in a cohort of patients followed at the GD Referral Centre of Porto Alegre, Rio Grande do Sul (RS), Brazil (CREDG-RS), from 2003 to 2023.

## 2 Material and methods

### 2.1 Study design

This retrospective cohort study performed descriptive analyses of patients with all types of GD followed at CREDG-RS between 2003 and 2023, focusing on morbidity and mortality data. CREDG-RS was established in 2003 at Hospital de Clínicas de Porto Alegre as the reference centre for GD in Rio Grande do Sul, Brazil. The analysis incorporated GD type, clinical manifestations, demographic characteristics (age, sex), genotype, treatment history, age at death, morbidities and causes of mortality. Ashkenazi Jewish ancestry was considered positive if reported by the patient or family. As a deeply-phenotyped cohort, all participants underwent comprehensive annual clinical evaluations. Genetic analysis identified *GBA1* variants using Sanger sequencing (ABI 3500 Genetic Analyzer) and massive parallel sequencing (Ion Torrent PGM™), following methodology previously described for Brazilian GD patients [8], [9], [10]. Regarding treatment, imiglucerase, velaglucerase alfa, taliglucerase alfa and miglustat are currently available through the Brazilian public health system.

### 2.2 Ethical considerations

The study was approved by the local Research Ethics Committee (CAAE: 2019–0219), complying with Brazilian human research regulations. Written informed consent was obtained from all participants or guardians.

### 2.3 Variables analysed

For deceased patients, age at death was used as the reference age; for surviving patients, current age (as of 2023) was considered. Clinical and demographic data were retrieved from medical records. Comorbidity was defined as any diagnosis, based on the International Classification of Diseases, 10th revision (ICD-10), that was not part of the natural history of GD. Accordingly, anemia, chronic pain, bone disease and strabismus (in GD3) were not counted as comorbidities. Owing to the short lifespan of GD2 patients, comorbidities were not analysed in this subgroup.

### 2.4 Statistical analysis

Continuous variables were expressed as median with interquartile range (IQR). Survival analysis was performed using Kaplan–Meier methodology with log-rank testing for group comparisons where applicable. Statistical significance was defined as two-tailed *p* < 0.05. All analyses were conducted using GraphPad Prism version 8.0 (GraphPad Software, San Diego, CA, USA).

## 3 Results

### 3.1 Demographic profile

Our cohort included 53 patients (male = 30; GD1 = 45, GD2 = 4, GD3 = 4). The median age was 45 years (IQR 25, range 15–75) for GD1, 0.42 years (IQR 0.63, range 0.1–0.8) for GD2, and 26 years (IQR 12.8, range 5–32) for GD3. Median time on treatment for GD1 was 14 years (IQR 8), for GD2 was 0.4 years (IQR 0.3), and for GD3 was 22 years (IQR 12). Only one individual reported Ashkenazi Jewish ancestry.

### 3.2 Treatment

No patient received ambroxol or haematopoietic stem cell transplantation. Among patients on ERT (45/53, 85%), two were receiving velaglucerase alfa, eight taliglucerase alfa and 35 imiglucerase at inclusion. Among patients on SRT, one was on eliglustat (post-study) and two on miglustat. Five patients received neither ERT nor SRT (GD1 = 1, GD2 = 4). The three SRT-only patients (all GD1) were distinct from the 45 ERT recipients, together accounting for all 53 patients; no patient received both SRT and ERT.

### 3.3 Comorbidities

Data on comorbidities are presented in Table 1.

**Table 1:** Comorbidities among Gaucher disease type 1 and 3 patients followed at the CREDG-RS (2003–2023, *n* = 49).

Comorbidity category	Comorbidity	GD 1 (*n* = 45) Median age (IQR) 45 years (25)	GD 3 (n = 4) Median age (IQR) 26 years (12.8)
Neurological	Chronic headache	10	0
	Parkinson's disease	1	1
	Epilepsy	0	2
Psychiatric	Depression	14	0
	Substance abuse	12	0
	Anxiety	5	0
	Bipolar disorder	1	0
	Psychosis	1	1
Cardiovascular	Hypertension	18	0
	Hypertrophic cardiomyopathy	3⁎	0
	Venous insufficiency	5	0
	Stroke	1	0
Neoplasia	Liver cancer	1	0
	Multiple myeloma	1	0
	Breast cancer	1	0
	Lung cancer	1	0
Hematological/Immunological	MGUS	3	0
	Pernicious anemia	1	0
	Cobalamin deficiency	1	0
	IgA vasculitis	0	1
	β-thalassemia	0	2⁎⁎
Metabolic/Endocrinological	Overweight/Obesity	22	1
	Insulin resistance/diabetes mellitus	5	0
	Malnutrition	2	2)
	Polycystic ovary syndrome	1	0
	Hypothyroidism	1	0
Musculoskeletal	Fibromyalgia syndrome	2	0
	Psoriasis	1	0
	Carpal tunnel syndrome	1	0
Gallbladder	Cholelithiasis	12	1
Gastroenterological	Gastritis	5	1
	Gastroesophageal reflux disease	4	0
	Diverticulosis	1	0
	Ulcerative proctitis	1	0
Hepatic	Steatosis	2	1
	Chronic hepatitis B	2	0
	Cirrhosis	1	0
Nephrological	Horseshoe kidney	1	0
	Chronic kidney disease	1	0
	Nephrolithiasis	1	0
Ophthalmological	Pterygium	2	0
	Cataract	2	0
	Glaucoma	1	1
	Ocular toxoplasmosis	1	0
	Strabismus	1	0
	Retinal disease	1	0

⁎ Hypertrophic cardiomyopathy presented by 3 GD1 patients is related to a heterozygous pathogenic variant p.Arg21Leu in the *TPM1* gene.

⁎⁎ Two GD3 patients who have β-thalassemia are siblings. MGUS: monoclonal gammopathy of undetermined significance.

In GD1 patients, the most prevalent comorbidities were psychiatric (*n* = 32/45, 71%), metabolic/endocrinological (*n* = 31/45, 69%) and cardiovascular (*n* = 27/45, 60%). Gallbladder disease was common (27%, *n* = 12/45), with cholelithiasis as the main manifestation; 58% (*n* = 7/12) developed cholecystitis and underwent cholecystectomy. Notably, obesity and overweight affected 49% of patients (*n* = 22/45). Hypertrophic cardiomyopathy was identified in three siblings who carried the heterozygous pathogenic variant p.Arg21Leu in the *TPM1* gene; all three are asymptomatic and under regular cardiology follow-up. Psychiatric conditions were frequent, with depression and substance abuse being the most prevalent. GD3 patients presented a distinct profile, including malnutrition and short stature; two GD3 siblings had β-thalassemia.

### 3.4 Mortality

Nine patients (17%) died during the study period (GD1 = 4; GD2 = 4; GD3 = 1); the deceased patients are summarised in Table 2. Five of these cases have been reported elsewhere [11], [12], [13], [14], [15]. Kaplan–Meier analysis confirmed significantly better survival in GD1 than in the other subtypes (log-rank χ^2^ = 82.37, *p* < 0.0001), with median survival not reached (77% censored; Fig. 1). GD2 exhibited 100% mortality before 15 months (median survival, 5.5 months), showing the steepest survival decline (Gehan-Breslow χ^2^ = 80.14, *p* < 0.0001), whereas GD3 had 25% mortality (1/4 deaths at 27 years) with median survival not reached (75% censored), presenting an intermediate trajectory with a significant trend versus GD1 and GD2 (log-rank trend χ^2^ = 16.58, *p* < 0.0001). Comparative survival analysis revealed a mortality gradient: GD1 < GD3 < GD2 (HR 5.1, 95% CI 3.0–8.8 for GD2 versus GD1).

**Table 2:** Demographic and clinical characteristics of deceased patients in our GD cohort from 2003 to 2023.

Pt	Gender	GD subtype	*GBA1* variant 1	*GBA1* variant 2	Age at diagnosis (y)	Time on ERT (y)	Age at death (y)	ERT dose (IU/kg)	Cause of death	Follow-up duration (y)	Reference
1	M	1	p.Asn409Ser	p.Asn409Ser	64	3	67	Imi, 45	Multiple myeloma	3	This study
2	M	1	NA	NA	63	1	65	Alg, 30	Lung cancer	1	This study
3	M	1	p.Asn409Ser	p.Leu483Pro;Ala495Pro;Val499=	61	0	62	Not treated	Sepsis	0.5	This study
4	M	1	p.Asn409Ser	p.Leu483Pro;Ala495Pro;Val499=	50	13	65	Imi, 30	HCA	13	Starosta et al. 2019
5	M	2	p.Leu483Pro	p.Leu483Pro	0.4	0.16	0.7	Imi, 60	Sepsis	0.3	Vernet Machado Bressan Wilke et al. 2024
6	M	2	p.Leu483Pro	p.Leu483Pro;Ala495Pro;Val499=	0.1	0	0.2	Not treated	Respiratory failure	0.1	Basgalupp et al. 2021
7	M	2	p.Leu483Pro	p.Leu483Pro	0.1	0.7	1.2	Imi, 60	Respiratory failure	1	Schwartz et al. 2009
8	M	2	p.Leu483Pro	p.Leu483Pro;Ala495Pro;Val499=	0.1	0	0.1	Not treated	Liver failure	0.03	This study
9	M	3	p.Leu483Pro	p.Leu483Pro	1	26	27	Vela, 60	Sudden Death	19	Vairo et al. 2013

NA: not available, ERT: enzyme replacement therapy, Imi: imiglucerase, Alg: alglucerase, Vela: velaglucerase alfa, HCA: hepatocellular carcinoma.

**Fig. 1:**
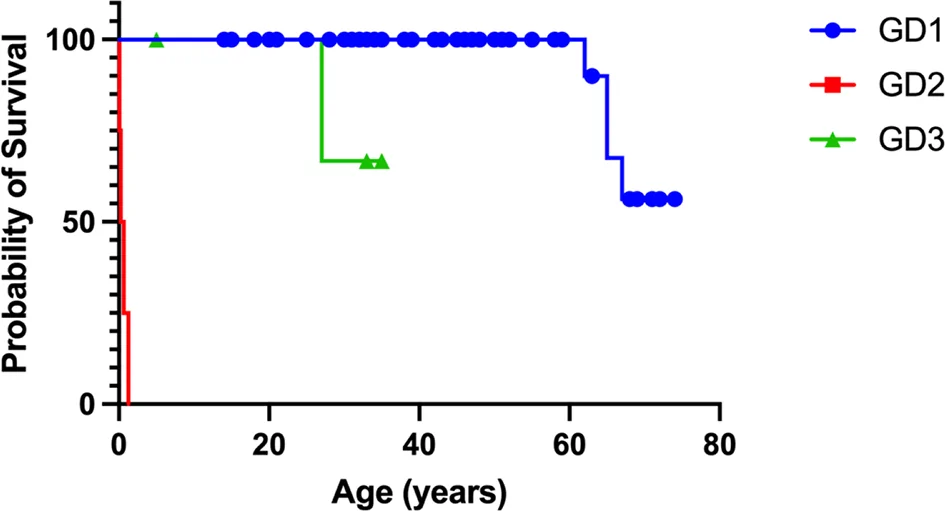
Kaplan-Meyer survival curves grouped analysis, including GD1 (blue), GD2 (red) and GD3 (green).

## 4 Discussion

To our knowledge, this is the first single-centre, deeply-phenotyped cohort study to characterise morbidity and mortality patterns in GD in a Latin American country, complementing the recent large-scale administrative-data analysis reported by Borin et al. [16]. In contrast to cohorts from African and Asian countries, in which GD3 predominates, our cohort was characterised mainly by GD1 patients. Regarding ethnic background, only one patient in our cohort reported Ashkenazi Jewish ancestry; broader ancestral data (e.g., European/African versus Native South American descent) were not systematically collected in this retrospective cohort, precluding a more detailed ethnic characterisation.

GD1 patients carried a substantial comorbidity burden, predominantly metabolic/endocrinological, cardiovascular and biliary. For the purposes of this analysis, metabolic/endocrinological comorbidities were operationally defined as any disorder of intermediary metabolism or endocrine regulation, including obesity/overweight, dyslipidaemia, type 2 diabetes, thyroid dysfunction, malnutrition and short stature. Within this spectrum, obesity and overweight were the most prevalent conditions, and cholelithiasis was also highly frequent. Psychiatric disorders were common, with 71% of GD1 patients diagnosed with depression, anxiety or substance abuse, underscoring the critical yet often overlooked mental-health component of managing a chronic rare disease. The high prevalence and clinical heterogeneity of these comorbidities also raise concerns about the potential for drug–drug interactions in adults with GD1 [17]. A notable hematological finding was the prevalence of Monoclonal Gammopathy of Undetermined Significance (MGUS) among GD1 patients (6%); one case progressed to multiple myeloma, which was the ultimate cause of death, in keeping with the well-recognised association between GD and monoclonal gammopathies [18]. In contrast, the aggressive natural history of GD2 precluded the development of any acquired comorbidities.

Compared with a Brazilian cohort of healthcare workers — in whom the most prevalent comorbidities were hypertension (31.9%), obesity/overweight (15.1%), pulmonary disease (12.9%), diabetes (10.4%) and depression (11.7%) [19] — our GD cohort showed a substantially higher overall comorbidity burden. Hypertension occurred at a similar prevalence in both groups, but obesity/overweight, depression and substance abuse were markedly more frequent among our patients. Notably, this comorbidity burden was not matched by a proportional mortality risk within our sample: cardiovascular and other comorbidities were frequent but were not associated with increased mortality in this cohort (data not shown). Instead, mortality was significantly elevated in patients with specific severe oncological, hepatic or neurological complications.

### 4.1 Mortality findings

In GD1, deceased patients shared several common features: they were male, three of the four carried at least one p.Asn409Ser variant in the *GBA1* gene, presented initially with hepatosplenomegaly, were of advanced age at diagnosis (median 62 years, range 50–64) and had received ERT for a short period — a factor that may have contributed to their mortality. The leading cause of death in this phenotype was malignancy, a finding consistently reported in the literature [18], [20], [21], [22], [23], [24]. Three of the four deceased GD1 patients died of cancer — hepatocellular carcinoma, multiple myeloma and lung cancer, respectively. An extensive risk-factor analysis of the patient who developed hepatocellular carcinoma identified GD as the primary precipitating factor, with no history of infectious hepatitis, alcohol abuse or iron overload. The patient who died of lung cancer had a long history of tobacco use; a synergistic effect between GD and environmental carcinogens cannot be ruled out. Compared with other GD cohorts, our patients had similar mortality aetiologies and survival despite their late diagnosis [24], [25], [26], [27].

### 4.2 Comparison with the Brazilian administrative-data cohort

Borin et al. [16] recently reported a 16-year retrospective analysis of 1234 Brazilian patients with Gaucher disease, assembled by linkage of the Unified Health System (SUS) administrative databases up to 2022. The inclusion criteria were the ICD-10 code E75.2 and the use of ERT/SRT. Their overall survival rates of 98.8%, 95.9%, 92.3% and 89.4% at 1, 5, 10 and 15 years under disease-specific therapy are consistent with the favourable long-term survival observed in our GD1 subgroup and support, at national scale, the effectiveness of the Brazilian public policy of universal access to ERT and SRT. More importantly, their multivariate Cox analysis identified Parkinson's disease (HR 12.2), hepatomegaly (HR 5.67), thrombocytopenia (HR 5.92) and splenectomy (HR 7.28) as the strongest predictors of mortality, with additional univariate signals for cancer (HR 15.4), diabetes (HR 26.4) and cardiovascular disease (HR 6.12). These population-level hazard ratios externally validate the clinical concerns raised by our cohort: the high prevalence of cardiovascular (60%) and metabolic/endocrine (69%) comorbidities in our GD1 patients marks conditions that carry measurable excess mortality when they occur; the malignancy-related mortality we observed in GD1 (3 of 4 deaths) is echoed by the very high hazard ratio for cancer in the SUS cohort; and the neurological burden we discuss in GD3 resonates with the central role that Parkinson's disease plays as a mortality driver in the population-level analysis.

The two designs are complementary rather than interchangeable. The ICD-10 code E75.2 does not distinguish among the three clinical phenotypes and neither does the SUS dispensation record, so the administrative cohort necessarily treats GD as a single entity, whereas natural history, life expectancy, causes of death and therapeutic response differ profoundly among GD1, GD2 and GD3. Our deeply-phenotyped cohort, in which every patient has a molecularly confirmed diagnosis, allowed subtype-specific survival estimates (log-rank χ^2^ = 82.37, *p* < 0.0001; HR 5.1, 95% CI 3.0–8.8 for GD2 vs GD1) and the identification of genotype–phenotype associations and cause-specific mortality — MGUS with progression to multiple myeloma, hypertrophic cardiomyopathy linked to a *TPM1* variant in three siblings, β-thalassemia co-segregating with GD3, SUDEP in GD3 and a substantial psychiatric burden — that no administrative dataset could detect. Taken together, Borin et al. provide a pharmacoepidemiological overview of GD in Brazil, whereas our study delivers the clinical counterpart, phenotypically and genotypically resolved by subtype in a long-term-followed referral cohort; both perspectives are needed to inform health-system priorities and to guide individual patient management in a heterogeneous disease.

### 4.3 GD2 prognosis and infant-mortality context

GD2 carries a grim prognosis. All GD2 patients in our cohort were male, and a genotype–phenotype difference was observed: patients with the compound heterozygous genotype p.[Leu483Pro];[Leu483Pro;Ala495Pro;Val499=] had a substantially shorter lifespan (median age at death, 2 months) than those with the homozygous p.Leu483Pro genotype (median age at death, 11.5 months). The severity of the compound heterozygous phenotype created a diagnostic dilemma, as some patients initially presented with only mild neurological impairment, making it difficult to distinguish acute GD2 from more attenuated neuronopathic forms. This diagnostic uncertainty led the clinical team to initiate ERT on compassionate grounds, in an attempt to alleviate systemic symptoms — a decision that also raised ethical questions about treating a uniformly fatal disorder. The survival curve for GD2 showed 100% mortality by 15 months (median, 5.5 months), and, within the limits of this small subgroup, ERT did not appear to modify this prognosis, in keeping with the neurological trajectory of the disease. Although we observed this genotype–phenotype relationship, whether GD2 prognosis can be reliably predicted from the *GBA1* genotype remains debated [3], [28].

The infant mortality profile of our GD2 patients also differed markedly from national data. In Brazil, infant mortality is predominantly concentrated in the neonatal period, with congenital anomalies, maternal factors, prematurity and perinatal infections as leading causes of death; in the post‑neonatal period, congenital anomalies and childhood infections predominate [29]. In contrast, our cohort showed a high incidence of multisystem failure — chiefly respiratory or hepatic — rather than deaths related to prematurity or congenital malformation.

### 4.4 GD3 mortality and SUDEP

Mortality in our young GD3 cohort was defined by a single case: a 27-year-old male who died of Sudden Unexpected Death in Epilepsy (SUDEP) despite lifelong ERT. A comparable pattern has been reported in an Egyptian cohort, in which similar unexplained deaths in GD3 were attributed to SUDEP [30]. The neurological trajectory of GD3 is heterogeneous, and long-term follow-up of treated patients has documented residual central-nervous-system, pulmonary and lymph-node involvement despite sustained visceral response to ERT [31], [32]. The overall severity of GD3 is further illustrated by our Kaplan–Meier analysis, in which its survival curve fell between those of GD1 and GD2 (*p* < 0.0001). This case underscores a key limitation of current therapy: although ERT may resolve non-neurological symptoms, it may not prevent neurologically-mediated mortality in the GD3 phenotype.

### 4.5 Overall analysis

This study illustrates the real-world challenges of managing GD in a middle-income country. Its strengths include a representative sample of GD patients from Southern Brazil, correlation of clinical findings with genotype, a distinctive analysis of the relationship between comorbidity burden and mortality, and a detailed description of mortality in both treated and untreated patients. Its main limitations are inherent to the retrospective cohort design and sample size: although our Kaplan–Meier analysis robustly captured subtype-specific survival patterns, the small GD2 and GD3 subgroups limit the precision of median survival estimates and preclude formal statistical modelling of survival, treatment effect or genotype–phenotype associations in these subgroups. Nevertheless, for a disease as heterogeneous as GD, deep phenotyping and molecular confirmation are the principal determinants of internal validity [2], and referral-centre cohorts remain a valuable complement to administrative datasets for characterising natural history. Our findings characterise a population with a substantial comorbidity burden that requires multidisciplinary care, in keeping with the broader supportive-care needs reported for adults with rare chronic diseases [33].

## 5 Conclusions

The comorbidity profile and causes of mortality in this GD cohort were phenotype-dependent. GD1 patients presented multiple comorbidities — mainly metabolic, cardiovascular and psychiatric — while mortality was driven by oncological and hepatic disease. In GD2, the natural history was dominated by the neurological phenotype; enzyme replacement therapy addressed only the visceral component of the disease, and all patients died before 15 months of age. GD3 patients presented mainly metabolic comorbidities, and the sole death was due to SUDEP in early adulthood. This retrospective study is representative of the population treated at our referral centre and is consistent with the international literature on GD from North American and European cohorts. It provides a coherent evidence base and a clinical baseline for future research, and supports the case for systematic, multidisciplinary surveillance of neurological, oncological, cardiovascular and metabolic comorbidities as a routine component of GD care in Brazilian public health settings.

## CRediT authorship contribution statement

**Lucas Ferreira Teixeira:** Writing – review & editing, Writing – original draft, Methodology, Investigation, Formal analysis, Data curation, Conceptualization. **Fabiano Poswar:** Writing – review & editing, Methodology, Investigation, Formal analysis. **Dévora Natalia Randon:** Writing – review & editing, Writing – original draft, Formal analysis, Data curation. **Amanda Pasqualotto:** Methodology, Investigation, Conceptualization. **Ida Vanessa Doederlein Schwartz:** Writing – review & editing, Supervision, Resources, Conceptualization.

## Ethics declaration

Written informed consent to take part in the study and to publish the article has been obtained from all participants or their legal representatives. The privacy rights of participants have been observed.

This study included organ or tissue donors. This study includes human biological material and consent was obtained by donors, or their next of kin or legal representatives, for use in this study and for publication of the article. The samples used in this research were not sourced from executed prisoners or prisoners of conscience.

This study was performed in compliance with relevant laws, regulatory frameworks and guidelines where the research took place. This study was approved by the Comite de Ética do Hospital de Clínicas de Porto Alegre (Approval No. 2019–0219).

The study was approved by the Research Ethics Committee of Hospital de Clínicas de Porto Alegre (CAAE 2019–0219), in accordance with Brazilian regulations on research involving human subjects. Written informed consent was obtained from all participants or their legal guardians.

## Declaration of generative AI and AI-assisted technologies in the writing process

During the preparation of this work the authors used Gemini 1.5 and Perplexity to improve readability and language. After using these tools, the authors reviewed and edited the content as needed and take full responsibility for the content of the publication.

## Funding

This work was supported by 10.13039/501100003593Conselho Nacional de Desenvolvimento Científico e Tecnológico (CNPq), Brazil, by the Fundo de Incentivo à Pesquisa e Eventos (FIPE) of Hospital de Clínicas de Porto Alegre, Brazil, and by Coordenação de Aperfeiçoamento de Pessoal de Nível Superior (CAPES), Brazil. The funders had no role in study design; in the collection, analysis or interpretation of data; in the writing of the report; or in the decision to submit the article for publication.

## Declaration of competing interest

The authors declare that they have no known competing financial interests or personal relationships that could have appeared to influence the work reported in this article.

## Data Availability

The data supporting the findings of this study are not publicly available because they include sensitive clinical information from patients with a rare disease, which could compromise participant privacy. De-identified data may be made available from the corresponding author on reasonable request and with the approval of the Research Ethics Committee of Hospital de Clínicas de Porto Alegre.
